# Imbalance in superoxide dismutase/thioredoxin reductase activities in hypercholesterolemic subjects: relationship with low density lipoprotein oxidation

**DOI:** 10.1186/1476-511X-11-79

**Published:** 2012-06-21

**Authors:** Paula Rossini Augusti, Amanda Roggia Ruviaro, Andréia Quatrin, Sabrina Somacal, Greicy Michelle Marafiga Conterato, Juliana Tanara Vicentini, Marta Medeiros Frescura Duarte, Tatiana Emanuelli

**Affiliations:** 1 Department of Biochemistry, Graduate Program on Biological Sciences/Biochemistry, Institute of Health Basic Sciences, Federal University of Rio Grande do Sul, Porto Alegre, RS, Brazil; 2 Campus Itaqui, Federal University of Pampa, Itaqui, RS, Brazil; 3 Department of Food Technology and Science, Integrated Center for Laboratory Analysis Development (NIDAL), Center of Rural Sciences, Federal University of Santa Maria, Santa Maria, RS, Brazil; 4 Departament of Health Sciences, Lutheran University of Brazil, Santa Maria, RS, Brazil

**Keywords:** Atherogenic index, Hypercholesterolemia, Oxidized low density lipoprotein, Superoxide dismutase, Thioredoxin reductase

## Abstract

**Background:**

There is a relationship among hypercholesterolemia, oxidative stress and inflammation in the atherogenesis. Thus, the objective of the present study was to assess paraoxonase (PON1), superoxide dismutase (SOD) and thioredoxin reductase (TrxR-1) activities and their relationship with lipids, oxidative stress and inflammation in subjects with different low density lipoprotein-cholesterol (LDL) levels.

**Methods:**

Serum lipids, highly sensitive C-reactive protein (hs-CRP), lipid and protein oxidation, oxidized LDL (LDLox) and LDLox autoantibodies (LDLoxAB) levels and enzymes activities were measured in a total of 116 subjects that were divided into the following groups according to their LDL levels: low-LDL group (LDL < 100 mg/dL, n = 23), intermediate-LDL group (LDL 100–160 mg/dL, n = 50) and high-LDL group (LDL > 160 mg/dL, n = 43).

**Results:**

The LDLox and hs-CRP levels increased in the high-LDL group (2.7- and 3.7- fold, respectively), whereas the intermediate and high-LDL groups had higher LDLoxAB (2.2- and 3.1-fold) when compared to low-LDL group (p < 0.05). Similarly, SOD activity, the atherogenic index (AI) and protein oxidation were also higher in the intermediate (1.3-, 1.3- and 1.2-fold) and high-LDL (1.6-, 2.3- and 1.6-fold) groups when compared to the low-LDL group (p < 0.05). Lipid oxidation and SOD/TrxR-1 ratio increased only in the high-LDL group (1.3- and 1.6-fold) when compared to the low-LDL group (p < 0.05). The SOD/TrxR-1 ratio was positively correlated to TBARS (r = 0.23, p < 0.05), LDLox (r = 0.18, p < 0.05), LDLoxAB (r = 0.21, p < 0.05), LDL (r = 0.19, p < 0.05) and AI (r = 0.22, p < 0.05). PON1 and TrxR-1 activities were similar among groups.

**Conclusions:**

Some oxidative events initiate when LDL levels are clinically acceptable. Moreover, hypercholesterolemic patients have an imbalance in SOD and TrxR-1 activities that is positively associated to LDL oxidation.

## Background

Atherosclerosis is the main underlying mechanism of leading causes of death, such as heart and brain disorders [1]. Hypercholesterolemia (HC), especially high levels of low density lipoprotein-cholesterol (LDL), seems to be an important risk factor accounting for severe atherosclerotic diseases, since LDL enters into the vessel walls by a concentration-dependent mechanism. Once into the endothelium, LDL suffers oxidative attack by reactive oxygen species (ROS) on its lipid and protein components, generating oxidized LDL (LDLox) [2]. Additionally, LDLox can initiate and enhance the inflammatory process, which plays a pivotal role in the development of atherosclerotic changes [3]. Accordingly, the level of highly sensitive C-reactive protein (hs-CRP), an inflammatory marker, is enhanced in patients with high levels of total cholesterol [3].

It has been suggested that high density lipoprotein (HDL) protects LDL particles from the oxidative process and subsequent inflammation [4]. This protective action of HDL is due to its associated enzyme paraoxonase 1 (PON1) [4]. Although PON1 activity is inversely associated to the incidence of coronary artery disease [5], there is no data on the behavior of PON1 during different stages of HC.

Since superoxide anion is thought to be one of the most important reactive oxygen species in the pathogenesis of atherosclerotic coronary artery diseases [6], extracellular superoxide dismutase (EC-SOD) plays a central role in cardiovascular antioxidant mechanisms. This SOD isoenzyme is mainly synthesized and secreted from vascular smooth muscle cells and macrophages and is the predominant arterial SOD isoform [7]. Thioredoxin reductase (TrxR-1) is a redox-active selenoprotein that efficiently regenerates oxidized thioredoxin protein (Trx-1) to its reduced form [8]. Reduced Trx-1 has an antioxidant function because it maintains the reduced state of many proteins [8]. Furthermore, TrxR-1 is overexpressed and released during oxidative stress and has been detected in plasma [9]. Although elevated Trx-1 levels have been found during HC and heart failure in humans [10,11], few reports are available on the behavior of TrxR-1 during coronary events or in patients with major risk factors, such as HC [12,13].

Considering the link among HC, oxidative stress and inflammation in the atherogenesis, this study reported the behavior of PON1 activity, as well as, the antioxidants SOD and TrxR-1 activities in the different stages of HC development. We also investigated the associations among the activity of these enzymes, oxidative stress and inflammatory markers and circulating lipids in the blood of subjects with different LDL levels.

## Results

Population characteristics are shown in Table 1. No significant difference was found among groups concerning age (p > 0.05). Total cholesterol (TC) levels increased significantly along with the increase in LDL levels among groups (p < 0.05). Triglycerides (TG) levels were also higher in the intermediate- and high-LDL groups when compared to the low-LDL group (p < 0.05), but no difference was observed between the high- and intermediate-LDL groups. In contrast, intermediate-LDL group had higher HDL levels than the other groups (p < 0.05). In addition, the atherogenic index (AI) increased in groups along with the increase in LDL levels (p < 0.05, Table 1).

**Table 1:** Characteristics of the studied groups

	**Groups (LDL-cholesterol levels)**		
	**Low-LDL**	**Intermediate-LDL**	**High-LDL**
	**(< 100 mg/dL)**	**(100–160 mg/dL)**	**(> 160 mg/dL)**
N	23	50	43
Sex (male/female)	12/11	26/24	16/27
Age (years)	55.9+2.3	54.1+1.9	55.3+1.5
	(34.0-77.0)	(29.0-87.0)	(31.0-75.0)
TC (mg/dL)	143.9+5.4^c^	212.1+3.5^b^	274.4+6.1^a^
	(84.0-188.0)	(138.0-270.0)	(226.0-449.0)
TG (mg/dL)	104.3+13.8^b^	143.9+13.1^a^	149.8+8.8^a^
	(20.0-317.0)	(32.0-430.0)	(67.0-300.0)
LDL (mg/dL)	81.3+3.6^c^	134.9+2.2^b^	202.9+5.8^a^
	(33.8-99.6)	(102.4-159.0)	(162.0-341.8)
HDL (mg/dL)	41.0+2.4^b^	48.5+1.9^a^	41.5+1.9^b^
	(23.0-72.0)	(21.0-95.0)	(20.0-91.0)
AI	2.7+0.2^c^	3.6+0.2^b^	6.1+0.4^a^
	(1.5-5.5)	(1.6-5.8)	(2.2-19.3)

Results are expressed as mean + SEM (minimum – maximum). ^a,b,c^ Values within the same line that do not share a common superscript letter are significantly different from each other (p < 0.05). TC = total cholesterol; TG = triglycerides; LDL = low-density lipoprotein; HDL = high-density lipoprotein; AI = atherogenic index.

One-way ANOVA revealed that patients from the high-LDL group showed significantly increased thiobarbituric acid reactive substances (TBARS) levels when compared to the low- and intermediate-LDL groups (p < 0.05, Table 2). On the other hand, protein carbonyl content increased in the intermediate- and high-LDL groups when compared to the low-LDL group (p < 0.05, Table 2), but no significant differences were found between the intermediate- and high-LDL groups. LDL levels affected LDLox and the levels of the inflammatory marker hs-CRP in the same way of TBARS levels, as it can be revealed by the nonparametric Kruskal-Wallis test (p < 0.05, Table 2). The LDLox autoantibodies (LDLoxAB) levels of patients from the intermediate- and high-LDL groups were significantly higher than those from the low-LDL group (p < 0.05, Table 2).

**Table 2:** Oxidative stress and inflammatory markers in patients with different LDL levels

	**Groups (LDL-cholesterol levels)**		
	**Low-LDL**	**Intermediate-LDL**	**High-LDL**
	**(<100 mg/dL)**	**(100–160 mg/dL)**	**(>160 mg/dL)**
TBARS (nmol MDA/mL)	4.9+0.3^b^	5.1+0.2^b^	6.3+0.3^a^
	(2.5-8.5)	(2.4-8.0)	(4.0-13.3)
Protein carbonyl content	1.2+0.1^b^	1.4+0.1^a^	1.9+0.2^a^
(nmol/mg of protein)	(0.2-2.9)	(0.7-4.9)	(0.5-6.7)
LDLox (mg/L)	0.27+0.06^b^	0.39+0.04^b^	0.73+0.06^a^
	(0.0-1.0)	(0.0-1.7)	(0.03-1.9)
LDLoxAB (mg/L)	10.3+2.1^c^	22.4+1.5^b^	32.1+1.4^a^
	(0.7-36.0)	(0.8-46.4)	(9.2-54.7)
hs-CRP (mg/L)	0.42+0.07^b^	0.81+0.16^b^	1.55+0.26^a^
	(0.1-1.2)	(0.1-7.9)	(0.1-7.9)

Results are expressed as mean + SEM (minimum – maximum). N = 23–50, as shown in Table 1. ^a,b,c^ Values within the same line that do not share a common superscript letter are significantly different from each other (p < 0.05). TBARS = thiobarbituric acid reactive substances; MDA = malondialdehyde; LDLox = oxidized LDL; LDLoxAB = LDLox autoantibodies; hs-CRP = highly sensitive C-reactive protein.

**Figure 1:**
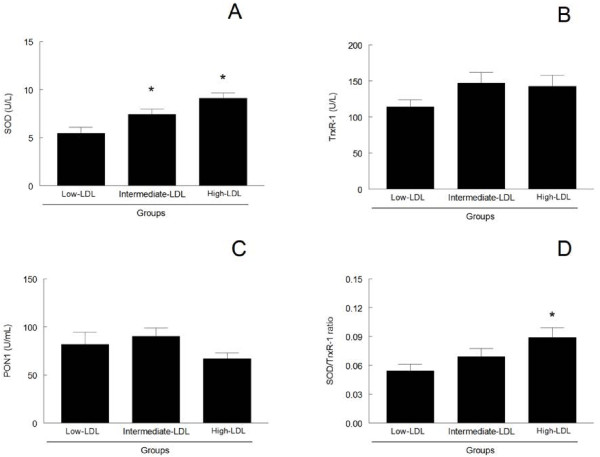
**SOD (A), TrxR-1 (B) and PON1 (C) activities and SOD/TrxR-1 ratio (D) of patients with different LDL levels.** Results are expressed as mean + SEM (n = 23–50, as shown in Table 1). *Significantly different from the low-LDL group (p < 0.05). SOD = superoxide dismutase; TrxR-1 = thioredoxin reductase 1; PON1 = Paraoxonase 1.

SOD activity was significantly higher in patients from the intermediate- and high-LDL groups compared to the low-LDL group (p < 0.05) and significant differences were found between the intermediate and high-LDL groups (Figure 1A). No significant differences were observed in TrxR-1 or PON1 activities among groups (p > 0.05, Figure 1B and 1C). Because TrxR-1 could be important to reduce the hydrogen peroxide (H_2_O_2_) that is generated during the dismutation of superoxide anion radical by SOD, we have also assessed the SOD/TrxR-1 ratio to verify a possible imbalance between these activities. The SOD/TrxR-1 ratio increased in the high-LDL group when compared to the low-LDL group, but no difference was observed between the low- and intermediate-LDL groups or between the intermediate- and high-LDL groups (p < 0.05, Figure 1D).

**Figure 2:**
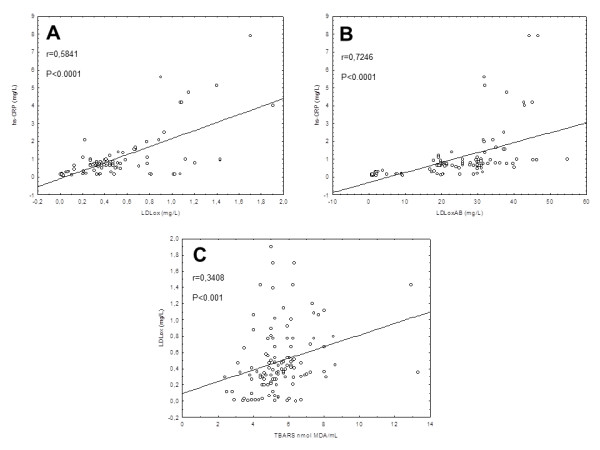
**Significant correlations between hs-CRP and LDLox (A), hs-CRP and LDLoxAB (B) and TBARS and LDLox (C).** TBARS = thiobarbituric acid reactive substances; MDA = malondialdehyde; LDLox = oxidized LDL; LDLoxAB = LDLox autoantibodies; hs-CRP = highly sensitive C-reactive protein.

LDL levels were positively correlated to TC, TG, TBARS levels, protein carbonyl content, LDLox, LDLoxAB and hs-CRP levels as well as to AI, SOD activity and SOD/TrxR-1 ratio (p < 0.05, Table 3). In contrast, LDL levels had no significant correlation with HDL levels, TrxR-1 or PON1 activities (p > 0.05, Table 3). A positive correlation was observed between TrxR-1and SOD activities (p < 0.05, Table 3). Despite the lack of additional associations of TrxR-1 with oxidative or inflammatory markers, SOD/TrxR-1 ratio was positively correlated with TBARS, LDLox and LDLoxAB levels, as well as, with the AI (p < 0.05, Table 3). Hs-CRP levels were positively correlated with LDLox as well as with LDLoxAB levels (p < 0.05, Figure 2A and 2B). In addition, we also found a positive correlation between TBARS and LDLox levels (p < 0.05, Figure 2C). PON1 activity had no correlation with oxidative or inflammatory markers in the present study (p > 0.05, Table 3).

**Table 3:** Associations among lipid levels, oxidative stress and inflammatory markers

**Parameters**	**Correlation coefficients (R)**			
	**LDL**	**PON1**	**TrxR-1**	**SOD/TrxR-1**
TC (mg/dL)	0.92*	−0.03	0.12	0.15
HDL (mg/dL)	−0.11	0.10	0.04	−0.09
TG (mg/dL)	0.21*	−0.09	0.002	0.06
Hs-CRP (mg/L)	0.46*	−0.06	−0.03	0.11
Protein carbonyl (nmol/mg of protein)	0.27*	0.05	0.08	0,00
TBARS (nmol MDA/mL)	0.33*	0.06	−0.05	0.23*
LDLox (mg/L)	0.49*	−0.02	−0.11	0.18*
LDLoxAB (mg/L)	0.66*	−0.04	−0.04	0.21*
AI	0.74*	−0.09	0.05	0.19*
SOD (U/L)	0.41*	−0.06	0.18*	-
TrxR- 1 (U/L)	0.10	0.05	-	-
SOD/TrxR-1	0.19*	−0.06	-	-
PON1 (U/mL)	−0.03	-	-	-

TC = total cholesterol; TG = triglycerides; TBARS = thiobarbituric acid reactive substances; MDA = malondialdehyde; SOD = superoxide dismutase; TrxR-1 = thioredoxin reductase 1; PON1 = Paraoxonase 1; LDLox = oxidized LDL; LDLoxAB = LDLox autoantibodies; hs-CRP = highly sensitive C-reactive protein; AI = atherogenic index.

## Discussion

Elevated plasma cholesterol levels play a dominant role in cardiovascular diseases [1] as can be evidenced by the occurrence of atheroma in the aorta of hypercholesterolemic rabbits [14]. Accordingly, the AI, which reflects the relationship between the non-HDL cholesterol fraction and the HDL cholesterol fraction and is considered a possible indicator of a predisposition to heart disease [15], was directly correlated to the increase in LDL levels. The injury theory of atherosclerosis holds that circulating LDL accumulates at susceptible sites where the oxidation of its protein and lipid components takes place, generating LDLox [2]. LDLox, in turn, participates in the inflammatory processes, contributing to lesion progression [3]. Our results corroborate this theory, since elevated LDLox levels were found in patients from the high-LDL group. Accordingly, lipid oxidation (TBARS) was also increased in patients from the high-LDL group and we observed a positive correlation between TBARS and LDLox, reinforcing that TBARS levels are at least partially associated to LDLox levels. In contrast to lipid oxidation, protein oxidation seems to start even when LDL levels are considered clinically acceptable, because the protein carbonyl content was increased in the intermediate-LDL group. The delay in the increase of LDL oxidation in the intermediate-LDL group may be related to a protective effect of LDLoxAB that was increased in this group compared to the low-LDL group. This idea is supported by previous studies which demonstrated that even minor modifications of native LDL render it immunogenic and that humoral antibodies are specific for the modifications to apo B [16,17]. These findings indicate that circulating antibodies respond to oxidative modifications in the protein moiety of LDL. In addition, humoral antibodies to modified LDL may redirect its site of degradation, primarily from plasma to the reticuloendothelial cells of the liver from rabbits [18]. Thus, LDLoxAB could enhance the removal of LDLox from serum and prevent its entrance into the arterial wall. In agreement, an inverse relationship between LDLoxAB and LDLox levels in healthy subjects is in line with the idea that LDLoxAB has a role in the clearance of LDLox from the circulation [19]. Moreover, at low rates of LDL oxidation, it is possible that an immune response might lead to an accelerated macrophage uptake of LDLox and thereby it may play a protective role. When the rate of oxidation is enhanced, however, the immune response might play a pathogenetic role, leading to accelerated macrophage uptake that overwhelms the capacity of the macrophage to handle the ingested LDLox [20]. Thus, we can speculate that the rate of oxidation in the intermediate-LDL group is low and LDLoxAB are still able to counteract LDLox, whereas in the high-LDL group this protection did not occur.

SOD activity increased along with the increase in LDL levels among groups and this increase occurred most likely to counteract the superoxide anion overproduction caused by HC [21]. The increase in SOD activity, along with the increase in protein carbonyl and LDLoxAB levels, even when LDL levels are considered clinically acceptable; indicate that oxidative stress is an early event in the evolution of hyperlipidemia.

The enzyme TrxR-1 along with protein Trx-1 has been recognized as an essential component for cellular redox control and antioxidant defense [8]. In this study, we demonstrated for the first time the behavior of serum TrxR-1 activity during different stages of HC in humans, although an elevation in serum reduced Trx-1 has already been described in patients with HC [10]. Additionally, we have recently demonstrated an increase in serum TrxR-1 activity in hypercholesterolemic rabbits [22]. However, in the present study, we observed no statistically significant differences in TrxR-1 activity among groups. The thioredoxin system (TrxR-1 and Trx-1) acts as a H_2_O_2_ scavenging system, reducing H_2_O_2_ to H_2_O [8]. Besides scavenging H_2_O_2_, TrxR-1 can indirectly maintain the reduced state of many proteins by regenerating oxidized Trx-1 to its reduced form [8]. Thus, we can speculate that the lack of changes in TrxR-1 activity in the intermediate-LDL group resulted in the increase of protein oxidation in this group. However, no association was found between TrxR-1 activity and protein carbonyl levels. Despite the unchanged activity of TrxR-1 among groups, we found a weak, but significant correlation between TrxR-1 and SOD activities, which could indicate a role for TrxR-1 in the removal of H_2_O_2_ produced by SOD. Interestingly, in the high-LDL group, SOD activity had a prominent increase over TrxR-1 activity, as measured by the increased SOD/TrxR-1 ratio. This imbalance in SOD/TrxR-1 activities suggests that TrxR1 activity could be lacking to efficiently remove the H_2_O_2_ produced by SOD. Because the SOD/TrxR-1 ratio was positively correlated with TBARS, LDLox and LDLoxAB levels, we can speculate that the imbalance in SOD/TrxR1 ratio could have deleterious effects in hypercholesterolemic subjects due to a peroxide overload. However, this proposal deserves further investigation because glutathione peroxidase and catalase, which are other important enzymes to remove H_2_O_2_ in plasma, were not evaluated in the present study. In fact, an imbalance between SOD and catalase activities has been well documented in various oxidative conditions [23,24], but to the best of our knowledge, this is the first report about an imbalance between SOD and TrxR-1 activity. Because the SOD/TrxR-1 ratio was associated with the AI, we can suggest a possible role of the SOD/TrxR-1 ratio as a marker of cardiovascular events during HC.

Similar to TrxR-1, the behavior of PON1 activity at different stages of HC has not been demonstrated yet. PON1 is believed to be responsible for the antioxidant effects of HDL [4] and its activity is inversely associated with the progression of atherosclerosis and the incidence of coronary artery disease [5]. In agreement, previous results from our group revealed diminished serum PON1 activity along with atherosclerotic plaque in the aorta of hypercholesterolemic rabbits [22]. Moreover, a large prospective study pointed low serum PON1 activity as an independent risk factor for coronary events in men at high risk of coronary heart disease [25]. However, we did not found differences in PON1 activity among the three studied groups or significant correlations between LDL levels and PON1 activity. This could be explained by an established balance between PON1 inactivation by oxidant species [26] and PON1 elevation in order to provide a protective mechanism against oxidant species and atherosclerotic plaque progression [27]. These differences in PON1 activity between previous studies and ours can be due the fact that studies concerning PON1 and cardiovascular diseases usually evaluated the relationship between serum enzyme activity and plaque formation [28] or cardiovascular events such as myocardial infarction [25]. Here, we evaluated the relationship between PON1 activity and another known predicting factor for cardiovascular diseases, the LDL levels. Moreover, PON1 seems not play a role in early atherosclerosis, although it may play a role in a later stage of cardiovascular diseases [29]. Since the atheroma formation was not evaluated in this study, we can speculate that unchanged PON1 activity and the lack of associations between PON1, LDL and LDLox levels are most likely because LDL levels evaluated in this study corresponded to early stages of atherosclerosis.

The involvement of oxidative stress in the initiation and progression of the inflammatory process is well documented [3]. Accordingly, we observed a significant correlation between LDLox and hs-CRP, which is an inflammatory marker. Moreover, serum hs-CRP levels were increased in the high-LDL group. Despite the changes in SOD and SOD/TrxR-1 ratio that accompanied the increase in LDL levels, no relationship was found between hs-CRP levels and antioxidant enzymes. This lack of correlation has already been described in the serum of patients with rheumatoid arthritis, although both parameters had been changed by the disease [30]. Although the extracellular reduced Trx-1 has proinflammatory effects by potentiating cytokine release from fibroblasts and monocytes [8], our results reveal that TrxR-1 seems to have no relationship with the inflammatory response during HC.

In conclusion, the present study demonstrated that some oxidative events initiate even when LDL levels are clinically acceptable. Moreover, hypercholesterolemic patients show an imbalance in SOD/TrxR-1 activities, which may play a role in the oxidative stress, because it is positively associated to LDL oxidation.

## Methods

### Study population

The population studied consisted of patients from LABIMED (Santa Maria, RS, Brazil). All subjects gave written informed consent to participate in the study. The work was carried out in accordance with The Code of Ethics of the World Medical Association and the protocol was approved by the Research Ethics Committee of the Federal University of Santa Maria (Protocol number: 23081.019182/2007-10). Subjects were divided into three groups according to the serum LDL levels, as follows: low-LDL group comprised subjects with LDL levels < 100 mg/dL (2.6 mmol/L); intermediate-LDL group comprised subjects with LDL levels ranging from 100 to 160 mg/dL (2.6-4.15 mmol/L); and high-LDL group comprised subjects with LDL levels > 160 mg/dL (4.15 mmol/L). These LDL ranges were selected according to the National Cholesterol Education Program [31], where LDL levels < 100 mg/dL are considered safe; between 100 and 160 mg/dL are clinically acceptable, but close to the limit; and LDL levels > 160 mg/dL are considered to be well above the safe limit for risk of cardiovascular diseases. Male and female subjects ageing 28 to 87 years-old were included in the study. Smokers, obese and hypertensive patients as well as patients with infectious diseases, metabolic syndrome and with blood glucose levels > 95 mg/dL were excluded from the study. Patients undergoing drug or vitamin treatment were also excluded from the study.

### Blood sample collection

Blood samples were collected after a 12-h overnight fasting by venous puncture into Vacutainer® (BD Diagnostics, Plymouth, UK) tubes with no anticoagulant. Blood samples were routinely centrifuged within 1 h of collection at 2500 × *g* for 15 min, and aliquots of serum samples were immediately used to assess TC, TG, HDL, LDLox, LDLoxAB, hs-CRP, TBARS levels and TrxR-1 activity. Then, serum samples were stored at −20°C for a maximum of 4 weeks before remaining measurements.

### Biochemical determinations

#### Lipid profile

TC and TG concentrations were measured by standard enzymatic methods using Ortho-Clinical Diagnostics® reagents on a fully automated analyzer (Vitros 950® dry chemistry system; Johnson & Johnson, Rochester, NY, USA). HDL cholesterol was measured after precipitation of apolipoprotein B-containing lipoproteins with dextran sulfate and magnesium chloride, as previously described [32]. LDL was estimated with the Friedewald equation [33]. The AI was calculated as (TC - HDL cholesterol)/HDL cholesterol as previously reported [34].

#### Oxidative stress markers

LDLox was determined by a capture ELISA according to the manufacturer instructions (Mercodia AB, Uppsala, Sweden) and as described before [35]. Serum samples were added to microplate wells coated with high affinity antibodies for LDLox. A peroxidase-conjugated antibody and tetramethylbenzidine (TMB) as substrate for peroxidase were used. The intensity of the yellow color, which is directly proportional to the LDLox concentration, was read at 450 nm. A standard curve was generated from standard LDLox. LDLoxAB was determined using ELISA as described by Wu and Lefvert [36]. Serum samples were added to microplate wells coated with high affinity antigen (LDLox). The methodology was similar to that used to quantify LDLox and the intensity of the yellow color that was directly proportional to the LDLoxAB concentration was read at 450 nm. A standard curve was generated from standard LDLoxAB. Lipid peroxidation, measured as TBARS levels, was assessed after the addition of 7.2 mM of butylated hydroxytoluene to prevent further oxidation. The reaction with thiobarbituric acid and extraction with *n*-butanol was performed as previously described [37] and the reaction product was determined at 535 nm using a standard curve of 1,1,3,3-tetraethoxypropane. Protein oxidation was assessed as protein carbonyl content based on the reaction of the carbonyl groups with 2,4-dinitrophenylhydrazine to form 2,4-dinitrophenylhydrazone [38]. Samples were read at 370 nm and carbonyl content was calculated using the molar absorption coefficient for aliphatic hydrazones (22,000 M^-1^ cm^-1^). SOD activity was determined at 480 nm using 50 mM glycine buffer, pH 10.2, and 1 mM epinephrine at 30°C [39]. SOD activity was expressed as the amount of enzyme that inhibits the auto-oxidation of epinephrine to adrenochrome by 50%, which is equal to 1 unit. TrxR-1 activity was determined using 5,5′-dithiobis (2-nitrobenzoic acid) (DTNB) and reduced adenine dinucleotide phosphate. The method is based on the reduction of DTNB, which is indicated by an increase in absorbance at 412 nm [40].

#### PON1 activity

PON1 activity was assessed by measuring the rate of paraoxon hydrolysis to yield p-nitrophenol, at 412 nm and 25°C [41]. The amount of p-nitrophenol generated was calculated using the molar extinction coefficient 17,000 M^-1^ cm^-1^ and 1 U of PON1 activity is defined as 1 nmol of p-nitrophenol generated per minute [41].

#### Inflammation marker

Hs-CRP was measured by nephelometry according to the manufacturer instructions (Dade Behring, Newark, DE, USA).

#### Statistical analysis

Data were analyzed by one-way analysis of variance (ANOVA) followed by unequal N HSD test when appropriate. Data that did not exhibit normal distribution were transformed (log or square root transformation) in order to meet ANOVA assumptions before analysis. When a variable was found not following normal distribution even after log or square root transformation, it was analyzed by the nonparametric Kruskal-Wallis ANOVA followed by the *post hoc* Dunn’s test when appropriate. The associations between variables were evaluated by Pearson’s correlation for variables that had normal distribution and by Spearman’s rank order correlation for variables that did not exhibit normal distribution. Results were considered significant when p < 0.05.

## Abbreviations

ANOVA: One-way analysis of variance; AI: Atherogenic index; HC: Hypercholesterolemia; HDL: High-density lipoprotein; Hs-CRP: Highly sensitive C-reactive protein; LDL: Low-density lipoprotein; LDLox: Oxidized low-density lipoprotein; LDLoxAB: Oxidized low-density lipoprotein antibodies; PON1: Paraoxonase; SOD: Superoxide dismutase; SOD/TrxR-1: Superoxide dismutase/ Thioredoxin reductase 1 ratio; TBARS: Thiobarbituric acid reactive substances; TC: Total cholesterol; TG: Triglycerides; TrxR-1: Thioredoxin reductase 1.

## Competing interests

The authors declare that they have no competing interests.

## Authors’ contributions

SS, AQ, ARR, GMMC, JV and PRA contributed to the experimental work. MMFD contributed to the experimental work, in particular in the quantification of inflammatory marker, oxidized low-density lipoprotein levels and oxidized low-density lipoproteins antibodies analyses. PRA and TE contributed in the design and planning of the study, as well as drafting and critical revision of the manuscript. All the authors contributed to the interpretation and discussion of results related to their part of the work and approved the final version of the paper.
